# Effect of magnesium infusion on thoracic epidural analgesia

**DOI:** 10.4103/1658-354X.76512

**Published:** 2011

**Authors:** Sampa Dutta Gupta, Koel Mitra, Maitreyee Mukherjee, Suddhadeb Roy, Aniruddha Sarkar, Sudeshna Kundu, Anupam Goswami, Uday Narayan Sarkar, Prakash Sanki, Ritabrata Mitra

**Affiliations:** *Department of Anaesthesiology & Chest Medicine, Institute of Postgraduate Medical Education and Research /SSKM Hospital, Bose Road, Kolkata, India*

**Keywords:** *Bupivacaine*, *epidural opioid*, *lung volume reduction surgery*, *magnesium infusion*, *post-thoracotomy pain*

## Abstract

**Introduction:**

Patients of lung volume reduction surgery (LVRS) having an ASA status III or more are likely to be further downgraded by surgery to critical levels of pulmonary function.

**Aim:**

To compare the efficacy of thoracic epidural block with (0.125%) bupivacaine, fentanyl combination and (0.125%) bupivacaine, fentanyl combination with adjunctive intravenous magnesium infusion for the relief of postoperative pain in patients undergoing LVRS.

**Methods:**

Patients were operated under general anesthesia. Thirty minutes before the anticipated completion of skin closure in both groups, (Group A and Group B) 7 ml of (0.125%) bupivacaine calculated as 1.5 ml/thoracic segment space for achieving analgesia in dermatomes of T4, T5, T6, T7, and T8 segments, along with fentanyl 50 *μ*g (0.5 ml), was administered through the catheter, activating the epidural block, and the time was noted. Thereafter, in patients of Group A, magnesium sulfate injection 30 mg/kg i.v. bolus was followed by infusion of magnesium sulfate at 10 mg/kg/hr and continued up to 24 hours. Group B was treated as control.

**Results and Analysis:**

A significant increase in the mean and maximum duration of analgesia in Group A in comparison with Group B (*P*<0.05) was observed. Total epidural dose of fentanyl and bupivacaine required in Group A was significantly lower in comparison with Group B in 24 hours.

**Discussion:**

Requirement of total doses of local anesthetics along with opioids could be minimized by magnesium infusion; therefore, the further downgradation of patients of LVRS may be prevented.

**Conclusion:**

Intravenous magnesium can prolong opioid-induced analgesia while minimizing nausea, pruritus, and somnolence.

## INTRODUCTION

Lung volume reduction surgery (LVRS) is a nonanatomic resection of lung tissue which removes approximately 20 to 30% of the poorly functioning emphysematous space occupying lung tissue from each lung. By reducing the lung size, the remaining lung and the surrounding muscles (intercostals and diaphragm) are able to work more efficiently, makes breathing easier, and thus the patients achieve a better quality of life. The improvement in lung function is greater with bilateral LVRS than unilateral, without any increase in morbidity or mortality. The bilateral approach is best conducted through a central incision.[1]

Pain management must be prophylactic, integral to surgery, and proactive than retroactive. LVRS is a modern procedure to which these principles apply.[2]

An effective regimen of postoperative analgesia not only shortens the time to recovery, but also helps in avoiding the pulmonary, hemodynamic, and metabolic complications of acute postsurgical pain.[3] Patients of LVRS having an ASA status III or more are likely to be further downgraded by surgery to critical levels of pulmonary function,[4] need special postoperative care as postoperative pain may lead to hypoventilation, areas of low ventilation to perfusion ratio, an increased intrapulmonary right-to-left shunt, atelectasis, functional disruption of respiratory muscle, temporary diaphragmatic dysfunction, inadequate cough leading to impairment of pulmonary secretion clearance, requirement of high FiO_2_, reduction in vital capacity and functional residual capacity (FRC).[5]

Thoracic epidural analgesia is considered by most to be the best and is effectively performed in various thoracic surgery centers.[6] Although opioids have been traditionally used as an adjunct,[7] they have their inherent dependent side effects like respiratory depression, diminished cough reflexes, changes in gastrointestinal motility, urinary retention, nausea, and vomiting.[8]

A comparative study conducted for post-thoracotomy analgesia in infants with bupivacaine and bupivacaine-fentanyl combination through thoracic epidural catheter showed that pain scores decreased significantly in the first 24 hours in the group with bupivacaine-fentanyl compared with bupivacaine alone.[9]

It has been concluded from previous studies that intravenous magnesium sulfate infusion with regional blockade decreases the analgesic requirement without enhancing the side effects of opioids or potentiating the side effects of regional blockade.[10–12]

So, an endeavor was made to compare the efficacy of thoracic epidural blockade with bupivacaine (0.125%), fentanyl 50 μg and bupivacaine (0.125%) combination, fentanyl 50 μg in combination with intravenous magnesium infusion for postoperative pain relief in adult patients undergoing LVRS.

### Aims and objectives

1. To compare the efficacy of thoracic epidural block with (0.125%) bupivacaine, fentanyl combination and (0.125%) bupivacaine, fentanyl combination with adjunctive intravenous magnesium infusion for the relief of postoperative pain in patients undergoing LVRS.
2. To note the complications if any.

## METHODS

This single center, prospective, randomized, controlled, double-blind study was carried out in the Department of Anaesthesiology of IPGME and R, SSKM Hospital, Kolkata, after obtaining permission from the Institutional Ethical Committee in the period between June 2007 and May 2010.

### Selection criteria

#### Inclusion criteria

A total of 60 adult patients of either gender aged between 35 to 60 years of ASA status II and III, scheduled for LVRS with ≥50% of predicted forced expiratory volume in 1^st^ second (FEV_1_), FEV_1_ /FVC (forced vital capacity), and mid-expiratory flow rate (MEFR), were included after obtaining informed consent from each patient.

#### Exclusion criteria

Patients of ASA status IV, with signs and symptoms of systemic infection or local sepsis, bleeding diathesis, coagulation abnormalities, diseases like diabetes mellitus, ischemic heart disease, hypertension, malignancy, renal or hepatic compromise or any major systemic illness, ongoing antiplatelet or anticoagulant therapy, hemodynamically unstable, hypersensitivity to the study drugs, spinal deformity, progressive neurological disease, mental retardation, psychiatric illness, pregnancy, or those who on awakening from general anesthesia complained of pain or not responding to vocal command were excluded from the study.

### Technique

Sample size was estimated using the PS (Power and sample size calculation version 2.1.30 February 2003). The sample size required for correctly rejecting the null hypothesis (of equal mean duration of effective analgesia—the primary end point) with a probability of 90% (i.e., power 0.90 or 90%) was calculated based on the following assumptions. Clinically important difference in mean duration of analgesia is 20 minutes with α = 0.05 or 5% (probability of type I error), σ = 35.26 min (within group SD obtained by a previous study by Kumar and Rajendran),[13] m = 1.2 (ratio of number of control to experimental patients); it was determined that 30 patients were required in each group (Group A and B).

#### Anesthesia technique

Following a detailed history and physical examination, breathing exercises and incentive spirometry were begun preoperatively. Fourth hourly nebulization with ipratropium bromide and salbutamol were started. All patients were instructed not to consume solid food after midnight on the day of surgery, but clear fluids were permitted till 4 hours before the scheduled time of operation. Patients were explained about the anesthetic technique, the blocks to be placed, the interpretation of visual analog scale (VAS), the method of postoperative analgesia, and to demand rescue analgesic at the onset of breakthrough pain (VAS>30 mm).

The patients were given alprazolam tablet 0.25 mg and omeprazole tablet 40 mg 2 hours before surgery. On arrival in the operating room, all the monitors were attached. Intravenous infusion of lactated Ringer’s solution was started; premedications of ondansetron injection 4 mg and glycopyrrolate injection 0.2 mg were given. Under strict aseptic precautions, on infiltration with local anesthetic (LA), an epidural 18G multiorifice catheter was placed 2 cm inside the epidural space through a 16G Tuohy epidural needle by paramedian approach at T5–T6 interspace. The epidural space was identified by loss of resistance technique using saline. A test dose of 3 ml of (1.5%) lignocaine with 1 : 200 000 adrenaline was administered through the epidural catheter on negative aspiration of CSF and blood to rule out accidental intrathecal or intravascular placement.[14a] Furthermore, the patients were put back to horizontal supine position. Fentanyl injection 2 μg/kg bodyweight i.v. was given 4 minutes before induction. After preoxygenation, the patients were induced with thiopentone injection 5 mg/kg i.v., or till the loss of eyelash reflex. Tracheal intubation was facilitated using double lumen tube after achieving full relaxation with vecuronium injection 0.1 mg/kg. Anesthesia was maintained using isoflurane 0.5 to 1% in 50% N_2_ O and O_2_, along with supplemental doses of vecuronium injection 0.02 mg/kg and fentanyl injection 0.5 μg/kg bodyweight i.v. top up doses, to maintain bispectral index between 40 and 60.

Thirty minutes before the completion of skin closure, all the 60 patients were randomized into two groups (Group A and B), generated by the statistical software ‘Microsoft Excel XPTM (2003).’

Thirty minutes before the anticipated completion of skin closure in both groups (Group A, Group B), 7 ml of (0.125%) bupivacaine calculated as 1.5 ml/thoracic segment space for achieving analgesia in dermatomes of T4, T5, T6, T7, and T8 segments,[14b] along with fentanyl 50 μg (0.5 ml), was administered through the catheter, activating the epidural block, and the time was noted. Furthermore, in patients of Group A, magnesium sulfate injection 30 mg/kg (10 ml) i.v. bolus was followed by infusion of magnesium sulfate (12 gm) in 50 ml normal saline at 2 ml/h (i.e.,10 mg/kg/h) and continued up to 24 hours. In patients of Group B, normal saline injection (10 ml) i.v. bolus was followed by infusion of normal saline at 2 ml/h and continued up to 24 hours. The nurse was engaged in the preparation of the infusions and the observer was blinded.

At the end of surgery, anesthetic agents were discontinued and neuromuscular blockade was reversed with neostigmine injection 50 μg/kg along with glycopyrrolate injection 10 μg/kg, after fulfilling the criteria for extubation.

Patients complaining of pain immediately after termination of general anesthesia were offered systemic opioid analgesia with tramadol injection 2 mg/kg bodyweight i.v. and excluded from the study.

The time interval between the activation of the epidural blocks in both the groups and the requirement of the first top up dose, when VAS >30 mm, was regarded as the effective duration of analgesia. Top up dose preparation contained bupivacaine (0.125% at 1.5 ml/thoracic segment space) along with fentanyl 50 μg (0.5 ml). All patients received top up epidural doses for the maintenance of postoperative analgesia for 72 hours.

Hemodynamic parameters, that is, noninvasive blood pressure monitoring for systolic blood pressure (SBP), diastolic blood pressure (DBP), mean arterial pressure (MAP), heart rate, urine output, respiratory rate, and patellar reflexes, were recorded from the immediate postextubation period to 24 hours postoperative period.

Incidence of untoward events like nausea, vomiting, pruritus, and respiratory depression were noted for the first 24 hours postoperative period, which were assessed using the evaluation scores noted in Table 1, as per scoring system of postoperative checkpoints.[15] Nausea was considered to be present if score was ≥2. Pruritus was considered present when score was ≥2. The patients were considered as participants of the study if alertness score was ≤2.

**Table 1:** Scoring system of postoperative checkpoints[15]

Nausea/vomiting	
No nausea	1
Complains of nausea but tolerable	2
Severe nausea needs medication	3
Pruritus (itching)	
No itching	1
Complain of itching but tolerable	2
Severe itching, needs medication	3
Respiratory depression	
None detected	1
Exist (RR <8/min)	2
Alertness	
Clear mentality	1
Good response to verbal command, but drowsy	2
Poor response to repeated verbal command	3

RR: Respiratory rate

The assessment was carried out by other anesthesiologists who were not involved in the care of the patients and blinded to the group assignment. Rescue medication was administered after any untoward event.

Primary outcome of the study was to determine the duration of effective analgesia (the time interval between the activation of the block and the first request for analgesic).

Secondary outcomes were VAS, complaints of pruritus, nausea, vomiting, respiratory depression, and hypotension (30% fall of blood pressure from baseline value) noted for the first 24 postoperative hours.

### Statistical analysis of data

All the data were entered into Excel Spreadsheet and analyzed using statistical software ‘SPSS and Statistica.’ Parametric data was presented as Mean (SD) and was compared by student t-test; nonparametric data by Mann Whitney U Test; and categorical data was compared by Fisher’s exact test. *P* value <0.05 was considered statistically significant.

## RESULTS

A total of 66 patients were needed for the study; as six patients complained of pain immediately after extubation (VAS >30 mm), they had to be excluded.

Table 2 shows the demographic profile (age, gender, BMI), the duration of surgery, VAS after extubation, and serum magnesium, urea, and creatinine levels to be comparable between the two groups (*P*>0.05).

**Table 2:** Comparison of demographic profile, duration of surgery, VAS after extubation, and serum magnesium, urea, and creatinine levels between the groups

	Group A	Group B	*P* value
Age (year)	47(10.6)	45 (10)	>0.05
Sex (M : F)	16 : 14	19 : 11	
BMI (kg/m^2^)	28 (5.7)	27 (4.1)	>0.05
Duration of surgery (min)	130 (25.55)	125 (20.25)	>0.05
VAS after extubation (mm)	24.28 (4.3)	25.45 (3.9)	>0.05
Serum magnesium level (mEq/l)	1.66 (0.22)	1.72 (0.18)	>0.05
Serum urea level (mg/dl)	26.33 (2.63)	29.12 (2.77)	>0.05
Serum creatinine level (mg/dl)	0.86 (0.12)	0.84 (0.14)	>0.05

BMI: Body mass index, VAS: Visual analog scale, Values are in Mean (SD) unless otherwise specified

The comparison of SBP, DBP, and MAP between the groups at various time points as seen in Table 3 were found to be comparable (*P*>0.05) in the immediate postoperative period and in the 1st, 2nd, 3rd, and 4th hours. However in the 5th hour, there was a significant difference (*P*<0.05) between the groups where Group B shows higher values. Also, though the readings were comparable in the 6th and 9th postoperative hours, the difference becomes statistically significant (*P*<0.05) in the 12^th^ hour, with Group B showing higher values.

**Table 3:** Comparison of systolic blood pressure, diastolic blood pressure, and mean arterial pressure (in mm Hg) between roups

	SBP	DBP	MAP
Immediate postoperative			
Group A	122.2 (12.12)	(9.64)	88.2 (10.2)
Group B	127.28 (9.84)	74.4 (9.98)	91.9 (11)
1 h postoperative			
Group A	117 (16.84)	(10.2)	87.2 (11.2)
Group B	123.64 (14.25)	72.24 (10.34)	92.24 (10.8)
2 h postoperative			
Group A	136.33(11.62)	(11.2)	95.33 (11.4)
Group B	137.5 (12.64)	78.22 (12.22)	97.83 (12.44)
3 h postoperative			
Group A	120.6 (11.32)	(11.3)	(11.34)
Group B	120 (11.62)	78.26 (11.4)	92.24 (11.66)
4 h postoperative			
Group A	122.8 (11.62)	(10.1)	88.1 (11.2)
Group B	121 (12.62)	75.24 (11.24)	93.6 (11.8)
5 h postoperative			
Group A	122.2 (11.12)	72.84 (9.83)	93.2 (10.8)
Group B	148.28 (9.84)#	96.8 (11.24)#	131.12 (10.6)#
6 h postoperative			
Group A	122.2 (11.22)	(10.2)	89.8 (10.66)
Group B	(9.94)	77.88 (11.84)	93.8 (11.2)
9 h postoperative			
Group A	(10.22)	(11.2)	(10.8)
Group B	(12.24)	(11.64)	93.2 (12.2)
12 h postoperative			
Group A	122.84 (10.2)	70.6 (9.8)	93.24 (11)
Group B	152.82 (10.34)#	98.8 (9.2)#	134.86 (11.8)#

# *P*<0.05, Values given as Mean (SD) unless mentioned otherwise, SBP: Systolic blood pressure, DBP: Diastolic blood pressure, and MAP: Mean arterial pressure

Table 4 compares the pulse (/min) and VAS (mm) between the groups at various time points. The readings are comparable in the immediate postoperative period, 1^st^, 2^nd^, 3^rd^, and the 4^th^ hours postoperative period in both the groups (*P*>0.05). The 5^th^ postoperative hour however shows significant difference (*P*<0.05), with Group B showing higher values. Furthermore, the 6^th^, 9^th^, and 12^th^ hour postoperative period readings are comparable. The 12^th^ hour reading shows an increase in pulse rates as well as VAS in both the groups.

**Table 4:** Comparison of postoperative pulse rates (/min) and VAS (mm) between the groups at various time points

	Pulse/min	VAS in mm
Immediate postoperative		
Group A	93 (8.2)	10.2 (3.1)
Group B	96 (9.88)	10.6 (4.4)
1 h postoperative		
Group A	80.44 (10.2)	11.6 (3.2)
Group B	88.22 (10.44)	12.4 (3.28)
2 h postoperative		
Group A	78.2 (9.4)	11.8 (2.8)
Group B	86.8 (9.12)	12.6 (3.98)
3 h postoperative		
Group A	79.82 (8.34)	11.4 (3.66)
Group B	84.8 (9.8)	12.88 (4.6)
4 h postoperative		
Group A	80.2 (8.8)	12.8(3.51)
Group B	85.6 (9.2)	22.8 (4.46)
5 h postoperative		
Group A	80.2 (8.22)	12.2 (3.44)
Group B	110.4 (8.2)#	38.88 (4.82)#
6 h postoperative		
Group A	82.1 (7.4)	15.2 (3.02)
Group B	89.6 (9.2)	33.56 (4.26)
9 h postoperative		
Group A	82.2 (7.4)	25.4 (3.98)
Group B	84.2 (9.2)	22.22 (3.4)
12 h postoperative		
Group A	85.2 (8.88)	30.88 (4.2)
Group B	88.2 (9.88)	32.2 (5.66)

# *P*<0.05, Values are in Mean (SD) unless specified, VAS: Visual analogue scale

**Figure 1:**
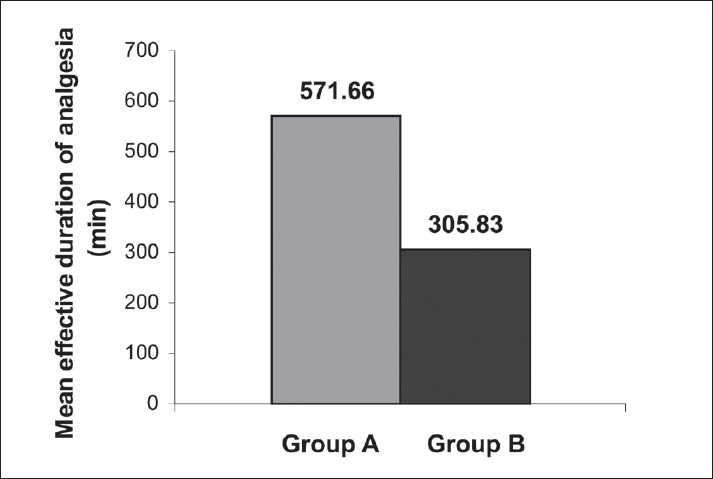
Comparison of mean effective duration of analgesia

A statistically significant (*P*<0.05) increase in the mean and maximum duration of analgesia (minutes) was found in Group A (571.66 [34.313]) in comparison with Group B (305.83 [77.313]) as seen in Table 5 and Figure 1.

**Table 5:** Mean effective duration of analgesia (min) in mean (SD)

Group	N	Mean (SD)
A	30	571.66 (34.313)
B	30	305.83 (77.313)
*P* value		<0.05

SD = Standard deviation

**Figure 2:**
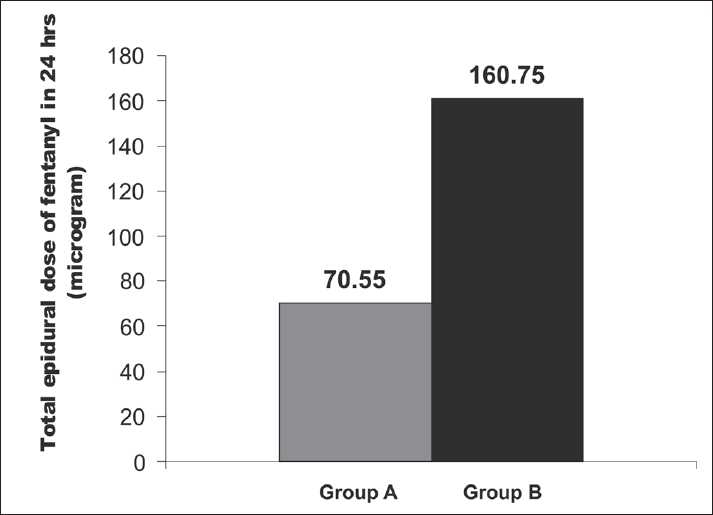
Comparison of total epidural dose of fentanyl (μg) in 24 hours

**Figure 3:**
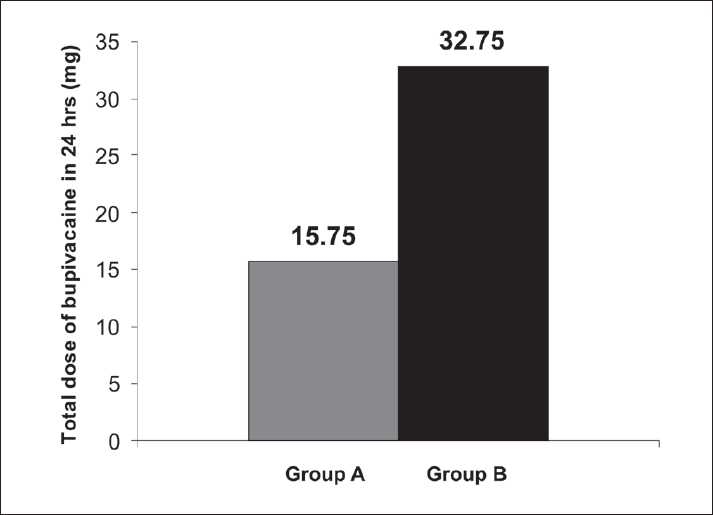
Comparison of total epidural dose of bupivacaine (mg) in 24 hours

The total intravenous dosage of fentanyl required in the intraoperative period is comparable in both the groups. However, the total epidural dose of required fentanyl (μg) is significantly higher in Group B (160.75 [20.75]) compared with Group A (70.55 [15.65]), which is statistically significant (*P*<0.05). Similarly, the total epidural dose of required bupivacaine (mg) is also higher in Group B (32.75 [5.67]) in comparison with Group A (15.75 [5.75]), which is also statistically significant. Pruritus was significantly less in Group A compared with Group B. The incidence of nausea and vomiting was also significantly less in Group A in comparison with the control. Respiratory depression was not observed in any patient. Patellar reflexes were normal in both the groups [Table 6] [Figures 2 and 3].

**Table 6:** Secondary outcome variables

	Group A	Group B	*P* value
Total i.v. dose of fentanyl in intraoperative period (μg)	110 (15.25)	115 (15.25)	>0.05
Total epidural dose of fentanyl in 24 h (μg)	70.55 (15.65)	160.75 (20.75)	<0.05
Total dose of bupivacaine in 24 h (mg)	15.75 (5.75)	32.75 (5.67)	<0.05
Patients with pruritus during first 24 h (%)	2 (7)	10 (33)	<0.05
Patients with nausea during first 24 h (%)	3 (10)	14 (47)	<0.05
Patients with vomiting during first 24 h (%)	1 (3)	9 (30)	<0.05
Patients with respiratory depression during first 24 h (%)	0	0	
Urine output in 24 h (ml)	1375.44 (178.88)	1299.26 (222.78)	>0.05
Absence of Patellar reflex (%)	0	0	>0.05

Values are in Mean (SD) unless otherwise specified

## DISCUSSION

Emphysema is a chronic, progressive disease of the lungs, most commonly caused by smoking. There occurs a breakdown in the walls of the air sacs of the lung which become abnormally enlarged, functioning poorly for oxygenation, also the small airways which carry air to and from the air sacs collapse during breathing, especially exhalation. These abnormally enlarged air sacs fill easily with air during inspiration but lose their ability to empty through the small airways during exhalation. This resembles airways obstruction. Thus, the term chronic obstructive pulmonary disease is used to describe emphysema. The problem of easy filling and poor emptying leads to progressive hyperexpansion of lungs.

Many people suffering from emphysema have portions of the lung more affected than others. This finding led to the development of a surgical approach for treatment, that is, LVRS, a procedure which removes portions of the poorly functioning, space-occupying tissue from each lung, which thereby improves the respiratory mechanics in severe emphysema by re-expansion of the functional lung tissue which was compressed by overdistended emphysematous alveoli. This restores the diaphragmatic mobility and improves the bellows function of the chest wall structures which work more efficiently making breathing easier and thus lead to a greater quality of life.

Ineffective postoperative pain management may lead to deep vein thrombosis, pulmonary embolism, coronary stress, atelectasis, pneumonia, poor wound healing, insomnia, and demoralization. Prevention and effective relief of acute pain may improve clinical outcomes, avoid clinical complications, reduce hospital stay, and thus save health care resources and improve quality of life.

Thoracic epidural anesthesia with LA and opioids appears to be the ideal means of achieving optimal intra- and postoperative analgesia with minimum central nervous system and respiratory depression. But it also has potential complications like hypotension, urinary retention, incomplete or failed block, and in rare instances paraplegia.[16]

A study by Tauzin-Fin *et al*. showed that intravenous magnesium infusion in radical prostatectomy under general anesthesia reduced the analgesic requirement.[10] Another study by Tramer and Glynn showed that in ambulatory inguinal hernia or varicose vein operations under general anesthesia with analgesic adjuvants, pretreatment with intravenous magnesium had no impact on postoperative pain or analgesic requirement.[11] Apan *et al*. showed that the addition of magnesium infusion as adjunct in spinal anesthesia reduced postoperative analgesic consumption.[12] This prospective randomized double-blind study including 60 adult patients between 35 to 60 years of ASA physical status II and III, scheduled for LVRS with ≥50% of predicted FEV1, FEV1/FVC, and MEFR, revealed a significant increase in the mean and maximum duration of analgesia (min) in magnesium recipients, that is, Group A [571.66 (34.313)] in comparison with control (i.e., Group B) (305.83 [77.313]), which was statistically significant (*P*<0.05). The total epidural dose of fentanyl required (μg) in Group A (70.55 [15.65]) was significantly lower in comparison with Group B (160.75 [20.75]) in 24 hours. Simultaneously, the total epidural dose of bupivacaine required (mg) in Group A (15.75 [15.65]) was also significantly (*P*<0.05) lower compared with Group B (32.75 [5.67]) in 24 hours. Associated complications related to opioids like pruritus, nausea, and vomiting were significantly lowered in the magnesium recipient group in comparison with the control group.

In thoracotomy patients, the motor effects of full concentrations of LA blockade result in increased FRC due to the caudad movement of the diaphragm and decrease in intrathoracic fluid volume which is beneficial, but the weakened inspiratory effort which accompanies is disadvantageous. Low-dose LAs come close to achieving an ideal situation by allowing for caudad movement of the diaphragm and recruitment of lung volume, without reducing the power of the intercostals necessary for coughing. In practice, motor block is reduced, almost to clinical insignificance, by the use of dilute solutions, without the loss of sensory block.[17]

Magnesium inhibits calcium entry into the cell by noncompetitive blockade of the N-methyl D-aspartate (NMDA) receptor.[18] Magnesium and NMDA receptor are thought to be involved in the modulation of pain.[19] The NMDA receptor antagonism inhibits induction and maintenance of central sensitization after nociceptive stimuli.[20] Magnesium is also a physiological calcium antagonist at different voltage-gated channels,[21] which may be important in the mechanisms of antinociception.[22] Thus, it potentiates the action of low-dose LAs and therefore may prevent the further downgradation of patients of LVRS with ASA status III or more to critical levels of pulmonary function.

Serum magnesium comprises only approximately 0.3% of total body magnesium, where it is present in three states—ionized (62%), protein bound (33%), and those bound mainly to albumin and complexed to anions such as citrate and phosphate. Equilibrium between tissue pools is reached slowly with a half-life varying between 41 and 181 days. Therefore, serum magnesium estimation may not provide representative information on the status of other stores.[23]

Post-thoracotomy pain management for LVRS thus poses a significant challenge to the anesthesiologist due to the high-risk patient population and nature of surgery. Management requires good understanding of the pathophysiology of the disease and the surgical procedure. Close co-ordination between the anesthesiologist and the critical care specialist with support staff is of paramount importance. In addition to pain management, nebulized bronchodilator therapy and aggressive chest physiotherapy should begin in the immediate postoperative period to maximize pulmonary function.

## CONCLUSION

Effective pain relief is vital to the success of LVRS and dictates the course of perioperative outcome. Considering the fact that thoracic epidural opioids have become the ‘Gold Standard’ for pain relief despite their potential side effects, coadministration of intravenous magnesium infusion may negate their undesired effects to reduce postoperative morbidity and mortality. However, the benefit of this therapy must be weighed against the potential adverse effects and the requirement of close observation. Thus, it may be concluded that intravenous magnesium can prolong opioid-induced analgesia while minimizing nausea, pruritus, and somnolence. However, we cannot state clearly whether this is the ideal dose or higher doses might produce fewer side effects while prolonging analgesia. Furthermore, it may be suggested that magnesium may be considered as one of the ingredients of multimodal analgesic stratagems in reducing the severity of post-thoracotomy pain.

### Limitations of the study

Perioperative magnesium assay was not done, and target-controlled infusion for accurate dose delivery of opioids for the maintenance of analgesia was not available.
